# In sight, in mind

**DOI:** 10.7554/eLife.35663

**Published:** 2018-03-01

**Authors:** Mariam Aly

**Affiliations:** Department of PsychologyColumbia UniversityNew YorkUnited States

**Keywords:** semantic memory, visual cognition, integration, fMRI, perirhinal cortex, ventral visual stream, Human

## Abstract

A region of the brain called the perirhinal cortex represents both what things look like and what they mean.

**Related research article** Martin CB, Douglas D, Newsome RN, Man LL, Barense M. 2018. Integrative and distinctive coding of visual and conceptual object features in the ventral visual stream. *eLife* **7**:e31873. doi: 10.7554/eLife.31873

When we look around at the world, we can appreciate what things look like and also what they are used for. For example, when we look at a couch, we see its long flat surface, its cushions, and its back. We also know that a couch is a good place to sit or nap. How does the brain represent, and integrate, these different kinds of information? This is a tricky question because these details are often related. A futon and a couch have similar functions and they look similar too. Because of this, it can be difficult to tell whether a given brain region codes for an object’s appearance (known as a percept) or its function (a concept).

Now, in eLife, Chris Martin, Morgan Barense and colleagues – who are based at the University of Toronto, Mount Allison University, the Rotman Research Institute, and Queen's University in Kingston – report how they have been able to tease out percepts and concepts in the brain (Martin et al., 2018). Their ingenious approach involved using the names of pairs of objects that look similar but have different functions, and other pairs with similar functions but different looks. For example, a tennis ball and a lemon are both roundish and yellow, but serve different purposes; a tennis ball and a tennis racket, on the other hand, do not look alike but are both involved in playing tennis.

Martin et al. asked over a thousand people to rate how much each pair of named objects looked alike, and another equally large group to describe conceptual features of those objects, for example, their function, or where they are typically found. For each pair of objects, these experiments gave one number that indicated the perceptual similarity of the objects, and a second number that indicated their conceptual similarity. Equipped with this information, Martin et al. could test different hypotheses of how percepts and concepts are represented in the brain.

One possibility was that some brain regions represent visual form (Martin and Chao, 2001) and others represent the function or meaning of objects (Patterson et al., 2007). An additional possibility, not exclusive of the first, was that some brain regions could simultaneously represent both (Barense et al., 2012a2012a; Clarke and Tyler, 2014; Murray and Bussey, 1999).

Functional magnetic resonance imaging (fMRI) examines brain activity on a moment-by-moment basis. Martin et al. used fMRI to observe how activity in different brain regions changed when individuals were shown the names of the objects, and did one of two tasks. In one task, individuals had to make judgments about what the object looked like; in the other task they had to make judgments about its conceptual features (e.g., what it is used for). Martin et al. could then look at the patterns of activity in different brain regions while people performed these two tasks, and relate those activity patterns to the ratings of perceptual and conceptual similarity they had obtained earlier (Kriegeskorte et al., 2008).

Martin et al. hypothesized that a region of the brain called the perirhinal cortex would represent what things looked like and what they meant. Prior studies have separately linked this brain region to both of these functions (e.g., Barense et al., 2012b; Wright et al., 2015), but could not disentangle perceptual and conceptual similarity. Having overcome that challenge with their experimental design, Martin et al. found that activity patterns in the perirhinal cortex did indeed reflect both perceptual and conceptual similarity. This result was obtained whether individuals were judging what objects looked like or what they meant, suggesting that this region of the brain may integrate percepts and concepts relatively automatically. Other regions of the brain represented either what things looked like or what they meant, but it was only the perirhinal cortex where both of these representations were integrated (Figure 1).

**Figure 1.**
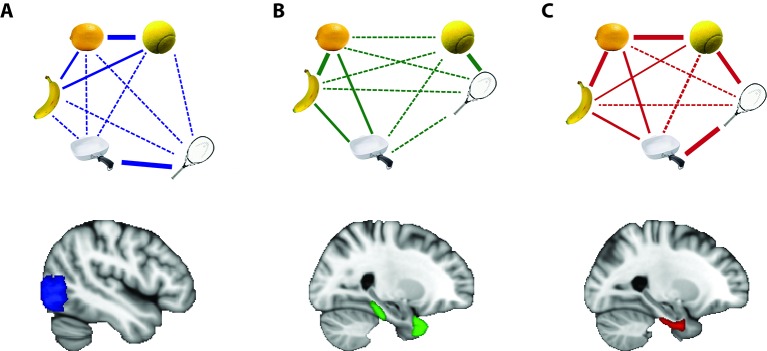
How visual and conceptual similarity are represented in different regions of the brain. Objects that are represented similarly in a given brain region are shown close together, with thick solid lines connecting them. Objects that are somewhat similar are shown at intermediate distance, with thin solid lines connecting them. Objects that are represented distinctly are shown further apart, with thin dashed lines between them. (**A**) A region of the brain called the lateral occipital cortex, shown in blue, represents objects that look alike – like a lemon and a tennis ball – in similar ways. (**B**) The temporal pole and parahippocampal cortex, shown in green, represent objects that are conceptually related – like a tennis ball and tennis racket – in similar ways. (**C**) The perirhinal cortex, shown in red, integrates these different kinds of information such that objects that are conceptually related or that look alike are represented in similar ways.

IMAGE CREDIT: Object images courtesy of Bainbridge and Oliva (2015).

Martin et al. have furthered our understanding of how we can perceive and understand objects, and their findings open some exciting avenues for future research. It remains unclear whether the exact same neurons in the perirhinal cortex represent both percepts and concepts at the same time, or if they are represented by distinct, but intermingled, populations of neurons. fMRI allows researchers to see at a general level which brain regions are active, but it cannot identify exactly which neurons are responding or how. Future studies that record from individual neurons will provide a complementary picture to this latest work.
